# The Traditional or Reverse Algorithm for Diagnosis of Syphilis: Pros and Cons

**DOI:** 10.1093/cid/ciaa307

**Published:** 2020-06-24

**Authors:** Daniel A Ortiz, Mayur R Shukla, Michael J Loeffelholz

**Affiliations:** 1 Department of Pathology, University of Texas Medical Branch, Galveston, Texas, USA; 2 Division of STD Prevention, National Center for HIV/AIDS, Viral Hepatitis, STD, and TB Prevention, Centers for Disease Control and Prevention, Atlanta, Georgia, USA

**Keywords:** syphilis, *Treponema pallidum*, diagnostic algorithms, serology

## Abstract

We reviewed relevant syphilis diagnostic literature to address the question “What diagnostic considerations should be taken into account when screening for syphilis using the traditional or reverse algorithm?” Improved laboratory diagnosis of syphilis is an important element of the effort to reduce syphilis rates. Screening for syphilis is performed using either a nontreponemal or treponemal test (part of the traditional or reverse algorithm, respectively). Both syphilis algorithms are used by laboratories. However, there are limited data on the performance and cost-effectiveness of the algorithms. An expert panel generated “key questions” in the laboratory diagnosis of syphilis. This paper pertains to the key factors that should be considered when deciding whether to screen for syphilis using either the traditional or the reverse algorithm. A systematic literature review was performed, and tables of evidence were created to address this question.

Screening for syphilis is performed using serological assays that detect treponemal and nontreponemal antibodies. The sequence in which these tests are performed differentiates the traditional from the reverse algorithm. In the traditional algorithm, a nontreponemal test (eg, rapid plasma reagin [RPR] or Venereal Disease Research Laboratory test [VDRL]) is used as the initial screen, and reactive samples are confirmed with a treponemal test. Conversely, the reverse algorithm uses a treponemal test for screening with reactive samples followed by a nontreponemal test. Discordant results between the treponemal screen and the nontreponemal test are resolved with a second confirmatory treponemal test (eg, *Treponema pallidum* particle agglutination) that preferably detects different antigens than the treponemal screen. Unfortunately, there is no gold standard for serologic syphilis testing, and therefore, all screening results must be correlated with clinical presentation for a diagnosis of syphilis.

According to a 2015 College of American Pathologists (CAP) survey, approximately 80% of laboratories perform the traditional algorithm and 20% perform the reverse algorithm when a single algorithm is offered at their facility [1]. A more recent review of CAP proficiency testing summary data showed a continuing trend toward increased use of the reverse algorithm. In 2019, 35.7% of CAP survey G (syphilis serology) participants reported use of a treponemal assay (authors’ unpublished data). The algorithm utilized is primarily driven by the volume of syphilis testing [2, 3]. Most nontreponemal tests are manual assays, so high-volume laboratories have chosen to adopt the reverse algorithm, due to availability of United States Food and Drug Administration (FDA)–cleared, automated treponemal platforms that perform high-throughput testing. Little guidance is provided to laboratories in choosing an algorithm, which is in part due to the limited data on the performance and cost-effectiveness of syphilis testing algorithms.

Improved laboratory diagnosis is crucial to curb the rise in syphilis infections. In 2016, the rate of reported syphilis in the United States reached an all-time high of 27.4 cases per 100 000 population, a > 2-fold increase since 2000 [4]. Increased incidence was seen across all stages of infection, and primarily attributed to men who have sex with men. However, all patient populations have been affected with increased rates reported in heterosexual men, women, and congenital infections. Despite improved laboratory methods, a syphilis diagnosis remains challenging to clinicians, especially with 2 algorithms currently recognized for diagnosis. To provide guidance on which algorithm to use, an expert panel reviewed the literature to determine the most effective approach based on laboratory workflow, cost-effectiveness, diagnostic accuracy, and public health impact.

## METHODS

A literature review was conducted based on the key question of factors to consider when deciding to screen for syphilis using either the traditional or reverse algorithm. Medline, Embase, Cumulative Index to Nursing and Allied Health Literature (CINAHL), Cochrane, and Scopus databases from January 2000 to June 2017 were queried with the following search terms: “Treponema pallidum” or “neurosyphilis” or “syphilis” and “sero-diagnose” or “serodiagnose” or “serology” and “test” or “exam” or “assay” or “screen” or “lab” or “diagnose” or “nontreponemal” or “treponemal” or “algorithm” or “antibodytiter” or “serofast” and yielded 4702 abstracts. Excluded from the search results were duplicate, animal model, non-English-language, non-FDA- cleared, and nonsyphilis abstracts resulting in 1851 total abstracts. The retrieved articles were then manually curated for potentially relevant papers using the terms “diagnosis,” “sero diagnosis,” “diagnostics,” “serology,” “serological test,” “treponemal,” “enzyme immunoassay,” “CLIA/CIA,” “IgG,” “IgM,” “TPPA,” “TPHA,” “nontreponemal,” “RPR,” “VDRL,” “laboratory screening,” or “algorithm” and yielded 251 abstracts. Meta-analysis, opinion, guidelines, letter to the editor, editorial, concepts, current trend/new trend, observations, guidance, reviews, non-FDA-approved tests, and international studies with non-FDA tests abstracts were excluded, finally giving 69 abstracts of which all full articles were pulled out and reviewed. Findings from the relevant article/data were summarized in form of a “table of evidence” for the key question. The data collected were presented to a group of syphilis experts convened in Atlanta, Georgia, 28–29 November 2017. Answers to the key questions were developed based on the tables of evidence, as well as expert opinion. Among the 69 articles in the “table of evidence,” a focus was placed on articles pertaining to the following: use of the traditional or reverse algorithm, evaluation of multiple FDA-approved assays, clearly stated gold standards (laboratory and/or clinical), signal strength-to-cutoff ratio, and cost-effectiveness.

## RESULTS AND DISCUSSION

Both the traditional and reverse syphilis algorithms are used by laboratories today. Low-volume laboratories typically utilize the traditional algorithm due to the low cost of the manual nontreponemal tests. The lack of automated nontreponemal platforms—until very recently—makes it difficult for high-volume laboratories to provide adequate turnaround times. High-volume laboratories have instead opted to perform the reverse algorithm as automated treponemal assays that increase throughput are widely available and FDA-cleared. With a shortage of medical laboratory technicians, automated systems also reduce labor costs and provide an ergonomic benefit by eliminating repetitive pipetting steps of manual assays. Recently, 3 automated nontreponemal platforms have been FDA-cleared (AIX1000, Gold Standard Diagnostics, Davis, California; BioPlex 2200 syphilis total and RPR, Bio-Rad Laboratories, Hercules, California; and ASI Evolution, Arlington Scientific, Springville, Utah), but there are limited peer-reviewed data evaluating their performance. As data become available, more high-volume laboratories may continue to use the traditional algorithm depending on the diagnostic accuracy of these automated assays and test cost.

Today, laboratories are constantly faced with pressure to increase output at a reduced cost, so test cost has become an important consideration when choosing an algorithm. However, data supporting the cost-effectiveness of either algorithm are sparse and conflicting. In 2 cost-analysis studies, Owusu-Edusei et al found that the traditional algorithm was more cost-effective in a low-prevalence setting (0.5%) and generated more cost savings in a high-prevalence setting (10%), which was largely attributed to less confirmatory testing compared to the reverse algorithm (Table 1) [5, 6]. Both analyses concluded that the amount of syphilis cases detected and treated was essentially the same when performing either algorithm in low- and high-prevalence settings. In contrast, Chuck et al found that a treponemal screen and confirmation was more cost-effective when screening prenatal and nonprenatal patient populations, with prevalence rates of 0.076% and 1.94%, respectively (Table 1) [7]. The Chuck et al analysis took into account more healthcare-associated costs (nurses, clerical services, infectious disease consultation) with a substantially higher cost for congenital syphilis ($16 017 vs $760.36) and neurosyphilis ($77 149 vs not assessed). The cost savings generated from preventing congenital and neurosyphilis favor the reverse algorithm due to more correct diagnoses (51 517 vs 51 510 in the prenatal population; 38 035 vs 37 876 in the nonprenatal population). However, the Chuck et al report assumed a sensitivity rate of 70.6% for RPR across all stages of infection. These conflicting reports suggest that the reverse algorithm may be more costly, but the socioeconomic impact of missing a syphilis diagnosis needs to be determined/standardized for cost-analysis studies to be useful.

**Table 1.** Summary of the Relevant Data

Syphilis Screening Algorithms				
Study, First Author and Year [Ref]	Study Design	Study Population	Gold Standard	Findings
Diagnostic implications				
Aktas 2005 [8]	Cross-sectional comparison of RPR and/or TPHA positive to Serodia TPPA, Syphilis ICE, and Enzywell TP	N = 1876 routine samples ≈4.5% prevalence	*Traditional algorithm* RPR followed by TPHA	23 RPR (+), 16 TPHA (+), and 84 both (+). 23 (1.23%) RPR biological false positives that did not confirm with TPHA, Serodia TPPA, the Murex Syphilis ICE, and the Enzywell TP tests. Lower false positives seen if TPHA used for screening 16 (0.85%). Agreement of TPHA (N = 124) with the Serodia TPPA, the Murex Syphilis ICE and the Enzywell TP tests were 96.7%, 100%, and 99.1%, respectively. Regardless of the treponemal test used as the confirmatory test, almost the same number of patients would have been diagnosed as having syphilis.
Aktas 2007 [9]	Cross-sectional comparison of FTA-ABS to TPHA, Mastaflourt FTA-ABS, Serodia TPPA, ICE Syphilis Detection Pack, and Enzywell TP	N = 122 FTA-ABS^+^ samples	FTA-ABS	Agreements of the FTA-ABS with the TPHA test, the TPPA test, ICE test, and TP test were 97.5%, 95.9%, 98.3%, and 98.3%, respectively. Agreements of the WB (n = 42) with TPHA, Serodia TPPA, Murex syphilis ICE, Enzywell TP, and FTA-ABS were 92.8%, 97.6%, 100%, 95.2%, and 92.8%, respectively. 2 FTA-ABS negative sera were positive by TPHA, TPPA, ICE, TP, and WB.
Angue 2005 [10]	Cross-sectional comparison of VDRL to Abbot Syfacard-R (RPR card test)	N = 2100 pregnant women 3% prevalence	VDRL	RPR: sensitivity 56.3% and specificity 96.5% High discordance rate between the RPR and VDRL
Berry 2016 [11]	Cross-sectional evaluation of signal-to-cutoff ratio	N = 665 (mixed population with 3.8% screen reactive rate)	Reverse algorithm (Bioplex IgG followed by RPR and TPPA)	99.3% of Bioplex IgG antibody index values of ≥ 8 were confirmed by TPPA, indicating that signal-to-cutoff ratio can be used in lieu of confirmatory testing. Total cost savings of $4825 annually.
Binnicker 2011 [12]	Cross-sectional comparison of 7 treponemal assays	N = 303 (203 routine and 100 previously tested samples)	FTA-ABS or consensus 4 of 7 treponemal tests	Agreements of the FTA-ABS with Bioplex 2200 Syphilis IgG, TPPA, Trep-Sure EIA, Trep-Check EIA, Trep-ID EIA, and Treponema ViraBlot IgG were 98.0%, 97.0% 95.4%, 97.7%, 98.4%, and 97.0%, respectively. Consensus 4 of 7 positive (panel) agreement for Bioplex 2200 Syphilis IgG, TPPA, Trep-Sure EIA, Trep-Check EIA, Trep-ID EIA, and Treponema ViraBlot IgG were 99.0%, 98.0% 95.7%, 98.7%, 99.3%, and 98.0%, respectively. Fastest TAT and throughput: Bioplex at 1.75 h for 100 samples and 514 samples for 9-h shift. Slowest TAT and lowest throughput: Trep-ID with 5.7 h for 100 samples and 158 samples in 9-h shift.
Binnicker 2012 [13]	Prospective, direct comparison of traditional and reverse algorithms	N = 1000 ≈1.5% prevalence	*Traditional and reverse algorithms* Discrepant results resolved by clinical data	*Traditional algorithm* 2 (0.2%) patients with possible latent syphilis missed 0 false reactive samples *Reverse algorithm* 6 (0.6%) false reactive samples
Bosshard 2013 [14]	Retrospective study evaluating IgM syphilis assays	N = 156 syphilis samples	Clinical symptoms and laboratory data (VDRL, TPPA, and Pathozyme Syphilis M Capture)	*Overall* n = *156* TPPA: sensitivity 100% and specificity 99.2% VDRL: sensitivity 83.3% and specificity 100% Pathozyme IgM: sensitivity 88.5% and specificity 96.0% *Primary syphilis* n = *59* TPPA: sensitivity 100% VDRL: sensitivity 61.0% Pathozyme IgM: sensitivity 89.8% *Secondary syphilis* n = *66* TPPA: sensitivity 100% VDRL: sensitivity 97.0% Pathozyme IgM: sensitivity 90.9% *Latent syphilis* n = *25* TPPA: sensitivity 100% VDRL: sensitivity 96.0% Pathozyme IgM: sensitivity 84.0%
CDC 2008 [2]	Retrospective study of 4 New York City laboratories using the reverse algorithm	N = 116 822 2.5% prevalence	Reverse algorithm: EIA followed by RPR	Among the 6548 EIA screen positive, 2884 (44%) were reactive and 3664 (56%) were nonreactive to the RPR test. 433/2079 (20.8%) of reactive EIA screens were non-reactive with a second treponemal assay. When TPPA was used as confirmation, 78/80 (98%) were reactive.
CDC 2011 [15]	Retrospective study of 5 laboratories (CA × 3, IL, and NY) using the reverse algorithm	N = 140 176 3 low-prevalence and 2 high-prevalence (Chicago and New York City) locations	Reverse algorithm: EIA/CIA followed by RPR	56.7% of reactive screens had a nonreactive RPR. 31.6% of reactive EIA screens were nonreactive with a second treponemal assay (FTA-ABS or TPPA). Among discordant sera, the rate of nonreactive confirmatory treponemal tests was 2.9 times higher in a population with low prevalence.
Creegan 2007 [16]	Cross-sectional study of primary syphilis	N = 106 primary syphilis	Clinical symptoms and dark field microscopy	VDRL: sensitivity 70.8% TPPA: sensitivity 85.9% RPR (n = 51): sensitivity 72.5% 12% of primary cases were missed by VDRL and TPPA. Similar performance between RPR and VDRL in primary syphilis. Traditional algorithm less sensitive in primary syphilis.
Dai 2014 [17]	Retrospective study evaluating signal-to-cutoff ratio of the Architect Syphilis TP assay	N = 8980 cancer patients 3.6% screen reactive rate	*European algorithm* Architect followed by TRUST and TPPA	100% of Architect reactive samples with a signal-to-cutoff ratio ≥ 9.9 were reactive by confirmatory testing.
Dang 2006 [18]	Cross-sectional evaluation of RPR, TPPA, and WB	N = 67 (20 primary or 47 secondary syphilis	Not defined	*Primary syphilis* n = *20* RPR: 12/20 (60%) TPPA: 18/20 (90%) WB: 20/20 (100%) *Secondary syphilis* n = *47* RPR, TPPA, and WB: 47/47 (100%)
Gratrix 2012 [19]	Retrospective review of data when changing from the traditional to the reverse screening algorithm	N = 243 969 routine samples	Clinical and laboratory data (RPR and TPPA)	Significant increase in the rate of late latent syphilis diagnoses after switching to the reverse screening algorithm. *Rate of late latent syphilis* Traditional algorithm: 0.07% (n = 97) Reverse algorithm: 0.14% (n = 137) No significant rise in cases of primary syphilis. Only 3 cases of primary syphilis would have been missed using the traditional algorithm.
Gratzer 2014 [20]	Retrospective medical records review	N = 52 suspected primary syphilis STD clinic	Clinical symptoms and laboratory data (FTA-ABS or RPR positive)	Trep-Sure: sensitivity 28/52 (53.8%) RPR: 40/52 (76.9%)
Gu 2013 [21]	Cross-sectional study of RPR and TRUST	N = 209 active syphilis stratified by stage N = 247 control sera	Clinical and laboratory data (EIA and TPPA)	RPR kit 1: sensitivity 98.7% and specificity 96.8% RPR kit 2: sensitivity 95.7% and specificity 97.6% TRUST kit 1: sensitivity 99.0% and specificity 98.0% TRUST kit 2: sensitivity 96.7% and specificity 96.8% *Nonreactive RPR and TRUST* Overall (n = 209): 1%–4.3% Primary (n = 30): 6.7%–10% Secondary (n = 92): 0%–1.1% Latent (n = 39): 0%–5.1% Neurosyphilis (n = 44): 0%–4.5% Biological false positives ranged from 2.0% to 3.2% with the highest rate seen in patients with SLE (10.4%).
Hunter 2013 [22]	Retrospective analysis of samples reactive only by Archictect Syphilis TP	N = 18 713 0.005% prevalence	*Reverse algorithm* Archictect Syphilis TP followed by RPR and TPPA	82 (9.4%) were reactive only by Archictect Syphilis TP. Chart reviews of 20 of these patients found that 11 (55%) had clinical or serological evidence of previous or subsequent syphilis.
Jost 2013 [23]	Cross-sectional comparison of 9 treponemal assays	N = 290 with 109 reactive and 181 nonreactive samples	TPPA	95 (85%) samples were nonreactive for the confirmatory RPR test. Agreements of the TPPA with FTA-ABS, INNO-LIA, LIASON, Trep-Sure, BioELISA, SD BIOLINE, CAPTIA IgG, Trep-ID, were 97.9%, 99.3%, 99.7%, 99.3%, 99.3%, 99.3%, 98.3%, and 100%. Analytical sensitivity in fold dilutions: FTA-ABS (4), CAPTIA IgG (8), INNO-LIA (16), TPPA (16), SD BIOLINE (64), Trep-ID (64), LIASON (128), BioELISA (128), Trep-Sure (512). Confirmatory test should have the same or better analytical sensitivity.
Knaute 2012 [24]	Retrospective study of response to treatment of syphilis	N = 264 (42% HIV positive and 13% with history of syphilis)	Clinical and laboratory data (VDRL, TPPA, and Pathozyme Syphilis M Capture)	VDRL sensitivity stratified by stage: primary 58%, secondary 100%, tertiary 100%, and latent 88%. TPPA sensitivity stratified by stage: primary 93%, secondary 100%, tertiary 100%, and latent 100%. Pathozyme IgM: sensitivity stratified by stage: primary 96%, secondary 91%, tertiary 62%, and latent 79%. For primary syphilis, the VDRL test should not be recommended as first-line screening test because of its lack of sensitivity.
Knight 2007 [25]	Cross-sectional evaluation of LIAISON vs CAPTIA Syphilis-G	N = 2645 (51 primary syphilis, 999 routine samples, 200 HIV, 200 pregnant, and 992 negative controls)	*Reverse algorithm* CAPTIA Syphilis-G followed by RPR Discordant results tested by TPPA	*LIAISON agreement with CAPTIA and reverse algorithm, respectively* Primary and secondary syphilis: 94.1% and 100% Routine samples: 93.2% and 98.7 HIV patients: 84.5% and 94.0% Pregnant patients: 98.0% and 100% Negative controls: 94.3% and 98.3% *Overall* LIASON: sensitivity 95.8% and specificity 99.1% 11 of 21 discordant results positive by TPPA.
Li 2016 [26]	Retrospective study evaluating signal-to-cutoff ratio of the Architect Syphilis TP assay	N = 20 550 1.3% screen reactive rate	*European algorithm* Architect followed by RPR and TPPA	54/267 (20.2%) reactive by RPR 185/267 (69.3%) reactive by confirmatory TPPA 117/117 (100%) of Architect-reactive samples with a signal-to-cutoff ratio >10 were reactive by confirmatory TPPA testing. Only 42/117 (35.9%) were reactive by RPR.
Loeffelholz 2011 [27]	Retrospective study evaluating signal-to-cutoff ratio of the Bioplex IgG	N = 6234 Screen reactive rate: incarcerated 7.5%, OB/GYN 1.6%, and delivery 2.6%	NA	An RPR titer of ≥ 1:2 was more likely to confirm by TPPA. Bioplex IgG antibody index > 8 provided highest specificity for TPPA confirmation.
Malm 2015 [28]	Cross-sectional comparison of RPR to VDRL	N = 729 (301 Guinea-Bissau, 201 Sweden, 30 performance panels, and 200 blood donors)	Macro-Vue RPR	VDRL: sensitivity 66.3% and specificity 98.5% High discordance rate between the RPR and VDRL
	Cross-sectional comparison of 4 FDA-cleared treponemal assays	N = 619 (301 Guinea-Bissau, 201 Sweden, 30 performance panels, and 200 blood donors)	TPPA and TrepSure Anti-Treponema EIA Screen	Similar sensitivity (98.7%–100%) and specificity (97.9%–100%) of FDA-cleared TPPA, TrepSure Anti-Treponema EIA Screen, and Liaison Treponema Screen. Architect Syphilis TP: sensitivity 99.5%–99.8% and specificity 87.6%–89.5%
Marangoni 2000 [29]	Cross-sectional comparison of WB, FTA-ABS, MHA-TP, and VDRL	N = 100 clinically characterized samples	Clinical data	WB agreement stratified by stage: primary 96%, secondary 100%, tertiary 100%, and latent 100%. FTA-ABS agreement stratified by stage: primary 65%, secondary 95%, tertiary 100%, and latent 100%. MHA-TP agreement stratified by stage: primary 65%, secondary 85%, tertiary 92%, and latent 100%. VDRL agreement stratified by stage: primary 66%, secondary 100%, tertiary 100%, and latent 100%.
Marangoni 2005 [30]	Retrospective and prospective study LIAISON compared to RPR, TPHA, WB	N = 2494 control sera N = 131 syphilis sera N = 96 analytical specificity N = 1800 prospective samples	Clinical and laboratory data	75 (2.90%) biological false-positive RPRs *Characterized syphilis sera* n = *131* LIASON: sensitivity 99.2% and specificity 99.9% EIA: sensitivity 95.4% and specificity 99.9% TPHA: sensitivity 94.7% and specificity 99.9% WB: sensitivity 100% and specificity 99.9% RPR: sensitivity 96.3% and specificity 97.1% *Prospective study* n = *1800* Overall agreement between the LIASON and WB (99.9%), EIA (98.7%), and TPHA (99.3%) were similar.
Mishra 2011 [31]	Retrospective review of data when changing from the traditional to the reverse screening algorithm	N = 3 092 938	Laboratory data	Confirmed positive rates increased by 10.3 per 100 000 population (*P* < .001) when switching to the reverse algorithm. Nonconfirmed RPR rate 0.13% Nonconfirmed EIA rate 0.26% 0.59% of EIA^+^/RPR^–^ patients converted to RPR^+^ within 2 months
Park 2011 [32]	Cross-sectional comparison of 6 automated treponemal assays	N = 155 FTA-ABS^+^/VDRL^+^ samples	*Reverse algorithm* (FTA-ABS followed by VDRL)	*Agreement, sensitivity, and specificity, respectively* Architect Syphilis TP: 99.2%, 96.8%, and 100% Cobas Syphilis: 99.8%, 99.4%, and 100% ADVIA Centaur Syphilis: 99.8%, 99.4%, and 100% HISCL Anti-TP: assay kit, 99.7%, 98.7%, and 100% Immunoticles Auto3 TP: 99.0%, 97.5%, and 99.6% Mediace TPLA: 98.0%, 98.1%, and 98.0% Automated immunoassays generally showed high sensitivities, specificities, and percentages of agreement compared to FTA-ABS.
Pope 2000 [33]	Cross-sectional comparison of MHA-TP to Serodia TPPA and CAPTIA Syphilis-G	N = 390 routine samples	MHA-TP	TPPA: agreement 97.4% Captia Syphilis-G: agreement 97.7%
Singh 2008 [34]	Cross-sectional study of primary and late latent syphilis cases that were initially nonreactive by RPR screening	N = 2166	*Traditional algorithm* RPR followed by MHA-TP or FTA-ABS	Primary syphilis: 224 (26%) nonreactive on initial RPR screening Late latent syphilis: 512 (39%) nonreactive on initial RPR screening
Tong 2014 [35]	Cross-sectional comparison of traditional, reverse, and ECDC algorithms	N = 24 124 ≈11.4% prevalence	Clinical symptoms and laboratory data (RPR, TPPA, and CIA)	Traditional algorithm: sensitivity 75.81%, specificity 99.98%, and accuracy 97.22%. Highest negative likelihood ratio (0.24), but lowest sensitivity for primary (75%) and tertiary (68%) syphilis; 71 biological false positives not confirmed by TPPA and CIA. Reverse algorithm: sensitivity 99.85%, specificity 99.82%, and accuracy 99.96%. 81 specimens were positive only by CIA. ECDC algorithm: sensitivity 99.38%, specificity 100%, and accuracy 99.93%. Both the reverse and ECDC had high sensitivity regardless of syphilis stage; 99.7% (665/667) of patients with RPR^–^/CIA^+^/TPPA^+^ were diagnosed with syphilis.
Wang 2016 [36]	Cross-sectional	N = 3962 routine samples ≈0.48% prevalence	Architect syphilis TP, RPR and TPPA Discrepant treponemal results resolved by WB	*Traditional algorithm* 19/3962 (0.48%) positive samples 11/3692 (0.28%) nonreactive by confirmatory by Architect and TPPA *Reverse algorithm* 20/3962 (0.66%) positive samples 5/3692 (0.13%) nonreactive confirmatory TPPA
		N = 36 000 routine samples ≈0.48% prevalence	*European algorithm* Architect Syphilis TP followed by TPPA Discrepant results resolved by WB	252/36000 (0.7%) positive by the Architect screen. 172/252 (68.3%) of Architect samples positive by TPPA. Among the 80 discrepant results, only 6 were reactive by WB. 100% of Architect reactive samples with a signal-to-cutoff ratio > 10 were reactive by confirmatory TPPA.
Wellinghausen 2011 [37]	Prospective and retrospective evaluation of LIASON to TPPA as a syphilis screen	Prospective N = 577 (318 pregnant) Retrospective N = 32 syphilis samples	Not defined	*Prospective study* LIAISON: sensitivity 100% and specificity 100% Architect Syphilis TP: sensitivity 100% and specificity 99.8% TPPA: sensitivity 100% and specificity 100% *Retrospective study* LIAISON, Architect, and TPPA all 100% sensitive
Wong/ 2011 [38]	Cross-sectional comparison of Trep-Sure EIA to VDRL and TPPA	N = 674 ≈9.4% prevalence	VDRL	Trep-Sure: sensitivity 87.7% and specificity 93.0% Trep-Sure EIA missed 6 VDRL^+^/TPPA^+^/WB^+^ specimens, and 6 (0.89%) reactive Trep-Sure EIAs were not confirmed by VDRL and TPPA. Among 269 specimens with a Trep-Sure EIA index score of ≥ 8.0, 268 (99.6%) were reactive by TPPA, indicating signal-to-cutoff ratio can be used to limit confirmatory testing. VDRL: 33 (4.9%) biological false positives
Yen-Lieberman 2011 [39]	Cross-sectional study to identify false-positive antibody results	N-142 Bioplex SyphG reactive samples ≈3% prevalence	Bioplex SyphG	Trep-Sure: agreement 77% Among the 27 RPR^+^ samples Trep-Sure had 100% agreement. All Bioplex SyphG samples above an antibody index value of 6.0, were confirmed by Trep-Sure indicating signal-to-cutoff ratio can be used for confirmation.
Young 2009 [40]	Retrospective and prospective study	N = 129 active syphilis stratified by stage N = 1107 prospective samples	Clinical and laboratory data	*Characterized syphilis sera* n = *129* Overall agreement with characterized syphilis sera: Architect CLIA 98.4%, Murex immune capture enzyme 86.0%, TPPA 98.4%, IgM EIA 86.8%, VDRL 83.7% Agreement with primary syphilis: Architect CLIA 97.5%, Murex immune capture enzyme 77.2%, TPPA 97.5%, IgM enzyme immunoassay 93.7%, VDRL 78.5% *Prospective study* n = *1107* Overall agreement between Architect and TPPA was 98.9% (1095/1107) Architect CLIA: sensitivity 100% and specificity 99.1% Murex immune capture enzyme: sensitivity 97.9% and specificity 99.9%
Zhang 2012 [41]	Cross-sectional evaluation of the analytical sensitivity of 5 treponemal assays (Bioplex IgG, LIAISON, Trep-Sure, Captia Syphilis-G, TPPA)	N = 10 (4 active and 6 past syphilis infections)	Laboratory data	Similar analytical sensitivities for Bioplex IgG, LIAISON, and CAPTIA Syphilis-G. Trep-Sure more sensitive by three 2-fold dilutions, and TPPA was the most sensitive by six 2-fold dilutions. The relative analytical sensitivities differ between treponemal assays and the confirmatory test should be at least as sensitive as the screening test.
Cost-effectiveness implications				
Chuck 2008 [7]	Simulation model comparing European and traditional algorithm	N = 89 647 (51 523 prenatal ≈0.076% prevalence and 38 124 routine ≈1.94% prevalence)	*European algorithm* EIA followed by INNO-LIA Traditional algorithm: RPR followed by TPPA or FTA-ABS	*Prenatal cohort* Cost: Can$9504 more using reverse algorithm Effectiveness: 1 new case and 6 correctly identified true negatives using reverse algorithm Cost-effectiveness ratio: Using the traditional algorithm will save Can$1358 *Routine cohort* Cost: Can$86 053 more using traditional algorithm Effectiveness: 3 new cases and 156 correctly identified true negatives using reverse algorithm Cost-effectiveness ratio: Using the reverse algorithm will save Can$541 When the cost for a false negative (Can$17 445) and false positive ($2962) are taken into account, the EIA followed by INNO-LIA is cost-effective in both prenatal and nonprenatal populations and will generate more correct diagnoses.
Owusu-Edusei 2011 [6]	Cohort decision analysis model to estimate cost and health outcomes of traditional and reverse algorithms	N = 200 000 with 1000 active and 1000 past infections 0.5% prevalence	NA	Net costs were $1.6 m for reverse algorithm and $1.4 m for traditional algorithm. Cost-effectiveness ratios were $1671 for the reverse algorithm and $1621 for the traditional algorithm per case treated. The cost-effectiveness of the traditional algorithm was lower as long as the treponemal test was > $4.10. Reverse algorithm identified 118 more cases leading to more follow-ups, which would result in identifying 1 additional case of tertiary syphilis. Reverse algorithm costs slightly more and leads to more unnecessary treatment.
Owusu-Edusei 2011 [5]	Cohort decision analysis model to estimate cost and health outcomes of traditional and reverse algorithms	N = 10 000 0.5% and 10% prevalence	NA	Low-prevalence setting: Reverse algorithm led to ≈2 times the number of confirmatory tests and was only cost-effective when the test was < $5.80. Traditional algorithm more cost-effective per adverse outcome ($1400 vs $1500). High-prevalence setting: Reverse algorithm led to ≈3 times the number of confirmatory tests and was only cost-effective when the test was < $1.80. Both algorithms detected the same number of syphilis cases in low- and high-prevalence settings. Reverse algorithm leads to overtreatment of uninfected patients.

Abbreviations: CA, California; Can$, Canadian dollars; CIA, chemiluminescence assay; CLIA, chemiluminescence immunoassay; ECDC, European Centre for Disease Prevention and Control; EIA, enzyme immunoassay; ELISA, enzyme-linked immunosorbent assay; FDA, United States Food and Drug Administration; FTA-ABS, fluorescent treponemal antibody absorption test; HISCL, high-sensitivity chemiluminescence enzyme immunoassay; HIV, human immunodeficiency virus; ICE, immune capture EIA; IgG, immunoglobulin G; IgM, immunoglobulin M; IL, Illinois; m, million; MHA-TP, microhemagglutination assay for Treponema pallidum antibodies; NA, not applicable; NY, New York; OB/GYN, obstetrics/gynecology; RPR, rapid plasma reagin; SLE, systemic lupus erythematosus; STD, sexually transmitted disease; TAT, turnaround time; TP, Treponema pallidum; TPHA, Treponema pallidum hemagglutination assay; TPLA, Treponema pallidum latex agglutination; TPPA, Treponema pallidum particle agglutination; TRUST, toluidine red unheated serum test; VDRL, Venereal Disease Research Laboratory; WB, Western blot.

Laboratories also should consider their patient population and syphilis risk when performing the traditional and reverse algorithm. Missing a syphilis diagnosis can have devastating effects particularly in a prenatal population where congenital syphilis is a concern. In high-risk patient populations (eg, sexually transmitted disease [STD] clinic patients, people with human immunodeficiency virus, men who have sex with men), routine screening at 3-month intervals, regardless of the algorithm used, is the most effective approach to identifying cases of early syphilis [42–45]. Screening with a nontreponemal test in the traditional algorithm detects cases of active syphilis, but reports have shown decreased sensitivity of the RPR and VDRL at detecting cases of primary and possibly latent syphilis, although many studies fail to differentiate latent from past treated syphilis based on treponemal confirmatory testing [2, 12, 15, 19, 31]. Screening with a treponemal test will identify more cases of syphilis, presumably past treated, but additional confirmatory testing drives costs up [8, 16, 18, 19, 30, 31, 40]. However, the signal strength (a semiquantitative value) of automated treponemal screening assays can predict when a confirmatory treponemal test will be reactive. Numerous studies have used a high signal strength (signal strength to cutoff ratio) in lieu of confirmatory treponemal testing to reduce unnecessary procedures or laboratory costs [11, 26, 27, 32, 36, 38, 39]. While each algorithm has a role depending on the patient population, these data suggest that a treponemal assay should be used to confirm a negative nontreponemal result if primary or latent syphilis is clinically suspected.

Several studies have attempted to compare the traditional and reverse algorithm, but they lack direct comparison because a nontreponemal and treponemal screen were not run in parallel. Only a single high-quality study directly compared the traditional and reverse algorithm by prospectively testing 1000 patient samples in a low-prevalence patient population with both algorithms [12]. The reverse algorithm in this study produced 6 false-reactive results, while the traditional algorithm had none. The false-reactive rate of the reverse algorithm (0.6%) was also consistent with a previous Centers for Disease Control and Prevention (CDC) report of 0.6% [15]. However, the traditional algorithm may have missed 2 patients with latent syphilis, although the definitive diagnosis of these patients was not determined. Recently, the CDC Division of STD Prevention and the Association of Public Health Laboratories have collaborated to developed a syphilis serum repository comprised of laboratory characterized, residual syphilis specimens with reported stages derived from laboratory submission forms, as submitted to public health laboratories [46]. These syphilis specimens serve as a resource for validating existing or new syphilis diagnostic tests and hence support public health, commercial or clinical institutions in the United States. Future studies should compare both algorithms prospectively or use clinically characterized samples stratified by stage of infection to effectively evaluate the performance of syphilis testing algorithms.

Use of the traditional or reverse algorithm is ultimately institution dependent based on patient population, test cost, volume, and workflow. The traditional algorithm is well suited for smaller laboratories with a low-test volume since manual nontreponemal screening assays are typically less expensive and have minimal effect on workflow. On the other hand, the reverse algorithm may be more appropriate for smaller laboratories serving a high-risk population, such as an STD clinic, where patients are more likely to be at risk for primary and latent syphilis that may be missed by the traditional algorithm. In larger laboratories, automated platforms improve workflow efficiency and provide a better turnaround time. Current data support the use of automated treponemal assays for screening, but this may change as more studies with automated nontreponemal assays become available. Regardless of the syphilis testing algorithm used, laboratory results should correlate clinically with patients’ symptoms and risk to make an accurate diagnosis. The laboratory can aid in the clinical decision process by collectively reporting all the laboratory results in a composite report that includes the algorithm, test methods, and interpretation.

## Notes

***Acknowledgments.*** The authors thank the members of the Centers for Disease Control and Prevention (CDC) sexually transmitted diseases laboratory group and external experts who developed or reviewed the key questions or supported the systematic literature reviews. The authors also thank our colleague, Dr John Papp, for insightful review of this manuscript.

***Supplement sponsorship.*** This article appears as part of the supplement National Coalition of STD Directors, sponsored by the CDC.

***Potential conflicts of interest.*** M. J. L. is an employee of Cepheid. All other authors report no potential conflicts of interest. All authors have submitted the ICMJE Form for Disclosure of Potential Conflicts of Interest. Conflicts that the editors consider relevant to the content of the manuscript have been disclosed.
